# Case of Instruments Lost

**Published:** 1911-07

**Authors:** 


### Case of Instruments Lost

DURING the life of the late Dr. William Warren Potter a case of surgical instruments which he had carried during his service as a surgeon in the civil war was stolen from his office by a sneak thief. It was later reported to Dr. Potter by a physician that he had purchased the instruments from a casual visitor and upon opening the case had found Dr. Potter's name engraved on the plate. It was Dr. Potter's intention to call for the case as indicated by conversation with associates and since his death it has been impossible to locate the physician. Information regarding the instruments will be gladly received at the JOURNAL office and its recovery will be welcome, since it is the intention to present it to one of the societies interested in the preservation of historic relics.

---

WHILE the opinion of President Taft (*Lancet Clinic*) may not be as important as his partisans would have people believe, still it is a pleasure to record his views on the medical profession and its achievements. In declining with regret an invitation to attend a banquet of the Philadelphia Medical Society, he remarked: "I may say one thing, and that is that we have real ground for national pride in the fact that England, France, Germany—Germany not so much so—and Holland have been engaged in the colonial business in the tropics for a hundred years, some of them two hundred years, and yet it remained for American physicians, and especially the physicians in the army, to discover more things in the ten years since the Spanish-American war; the discoveries of the American physicians since that time—what I may term the sequence of the war—were ample to justify the expenses of that war ten times over. It is a real record of achievement of a national character that every one who understands it must dwell upon with sincere pride.

---

In spite of the efforts of the authorities in all sections of the United States there will be the usual number of gunshot accidents during the celebration of July 4 this year and the medical profession, as usual, will be called upon to save the victims. The mortality in tetanus, the frequent result of firecracker and cartridge wounds, is largely decreasing owing to the use of tetanus antitoxin. In all cases of such wounds of celebration as have been mentioned it is urged that a prophylactic injection of tetanus antitoxin be used immediately.

---

The Ohio legislature has passed a bill prohibiting the indiscriminate distribution of medicines and medicinal preparations from door to door for advertising purposes. The bill has become a law by the signature of the governor and will be strictly enforced.

---

THE towns of Stockton and Charlotte in Chautauqua county have been placed under rabies quarantine by the state department of agriculture it having been determined that a dog which bit two men and a number of cattle was suffering from rabies.

---

A considerable sum of money was raised in Buffalo on June 10, District Nursing Day, by voluntary contribution of the public for the aid of the District Nursing Association.

